# Chip-Integrated Vortex Manipulation

**DOI:** 10.1021/acs.nanolett.3c00324

**Published:** 2023-03-14

**Authors:** Itai Keren, Alon Gutfreund, Avia Noah, Nofar Fridman, Angelo Di Bernardo, Hadar Steinberg, Yonathan Anahory

**Affiliations:** † Racah Institute of Physics, The Hebrew University, Jerusalem 91904, Israel; ‡ Department of Physics, University of Konstanz, Universitätstrasse 10, 78457 Konstanz, Germany; § Center for Nanoscience and Nanotechnology, Hebrew University of Jerusalem, Jerusalem 91904, Israel

**Keywords:** Vortex Manipulation, Topological Quantum Computation, Braiding, Scanning SQUID-on-Tip Microscopy

## Abstract

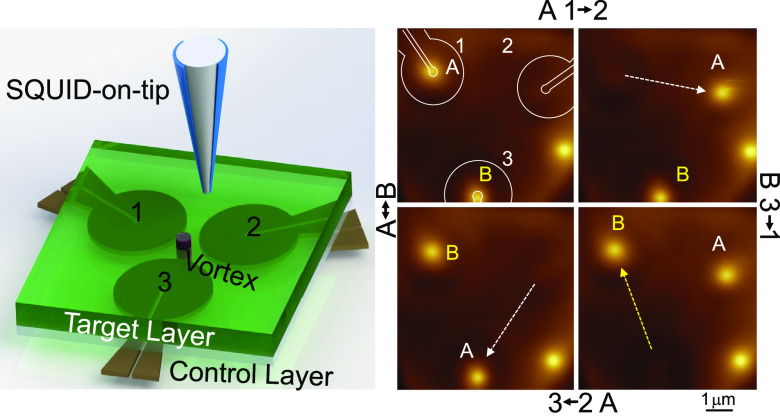

The positions of Abrikosov vortices have long been considered as means to encode classical information. Although it is possible to move individual vortices using local probes, the challenge of scalable on-chip vortex-control remains outstanding, especially when considering the demands of controlling multiple vortices. Realization of vortex logic requires means to shuttle vortices reliably between engineered pinning potentials, while concomitantly keeping all other vortices fixed. We demonstrate such capabilities using Nb loops patterned below a NbSe_**2**_ layer. SQUID-on-Tip (SOT) microscopy reveals that the loops localize vortices in designated sites to a precision better than 100 nm; they realize “push” and “pull” operations of vortices as far as 3 μm. Successive application of such operations shuttles a vortex between adjacent loops. Our results may be used as means to integrate vortices in future quantum circuitry. Strikingly, we demonstrate a winding operation, paving the way for future topological quantum computing and simulations.

Scalable single-vortex control has long been missing in the nanoscale superconducting device toolbox. Although superconducting classical computation is currently outperformed by silicon-based electronics, vortex based computation was proposed in low-temperature logic circuits^1^ and memory devices.^2−4^ Furthermore, the presence or the absence of an Abrikosov vortex within a Josephson device was suggested as a classical computation element.^5^ In quantum coherent circuitry, vortex removal from undesired positions could overcome vortex-induced dissipation and decoherence.^6−8^ In contrast, vortices could be used as carriers of topological quantum information. The operation of winding two vortices is considered an essential part of future topological quantum information processing.^9−14^ Previously, single-vortex manipulation was achieved by locally reducing the superconducting gap Δ, using current injection,^15,16^ heat^15,17,18^ or mechanical pressure.^19^ However, trapping vortices by reducing Δ is limited to a short-range, it can only attract vortices (and not repel them), and it drives the system out of equilibrium. Another avenue, is to exert a long-range Lorentz force which can either attract or repel vortices.^20−26^ These methods are not integrable on a chip, are not suitable for multivortex manipulation, and are limited by the scanning speed.

In this work, we develop a dynamic vortex control method. It is based on loop elements which may be placed above or below the target superconductor. A broad loop with a finely defined inner diameter combines the high current required to exert magnetic forces over long ranges, with a finely resolved pinning potential at the center. Use of a loop material with a low penetration length λ, locally expels vortices and creates an effective exclusion zone for vortices within the target superconductor. The loops are designed with a directional asymmetry, exerting a directional force along a predetermined vector.

Figure 1a presents the basic paradigm of the experiment, where we use a high-resolution SOT microscope^27,28^ to magnetically image the device, where the full vortex-control functionality is realized. The device consists of a bottom layer of 100 nm thick Nb superconducting loops fabricated by etching a Nb film using reactive ion etching (see the Methods section and Note 1 in the SI). A single loop, shown schematically in Figure 1b, has an inner diameter of 200 nm and outer diameter of 2.6 μm. A SEM image of the loops is shown in Figure 1c. We refer to these loops as the *control layer*. An Al_2_O_3_ layer (∼15 nm) is deposited on the control layer to electrically decouple the loops from the top superconducting layer where the vortices, which we want to manipulate, reside. Here we use a NbSe_2_ flake positioned using the standard polycarbonate (PC) flake transfer method. We refer to the latter as the *target layer*. The target layer is depicted schematically as a green square in Figure 1a and imaged in Figure 1c where the flake edges are outlined with a dashed black line. We emphasize that all vortices considered in this work thread solely the target layer. The loops are numbered for later reference (see Figure 1a,c,d). Detailed design considerations are discussed in Note 2 in the SI.

**Figure 1:**
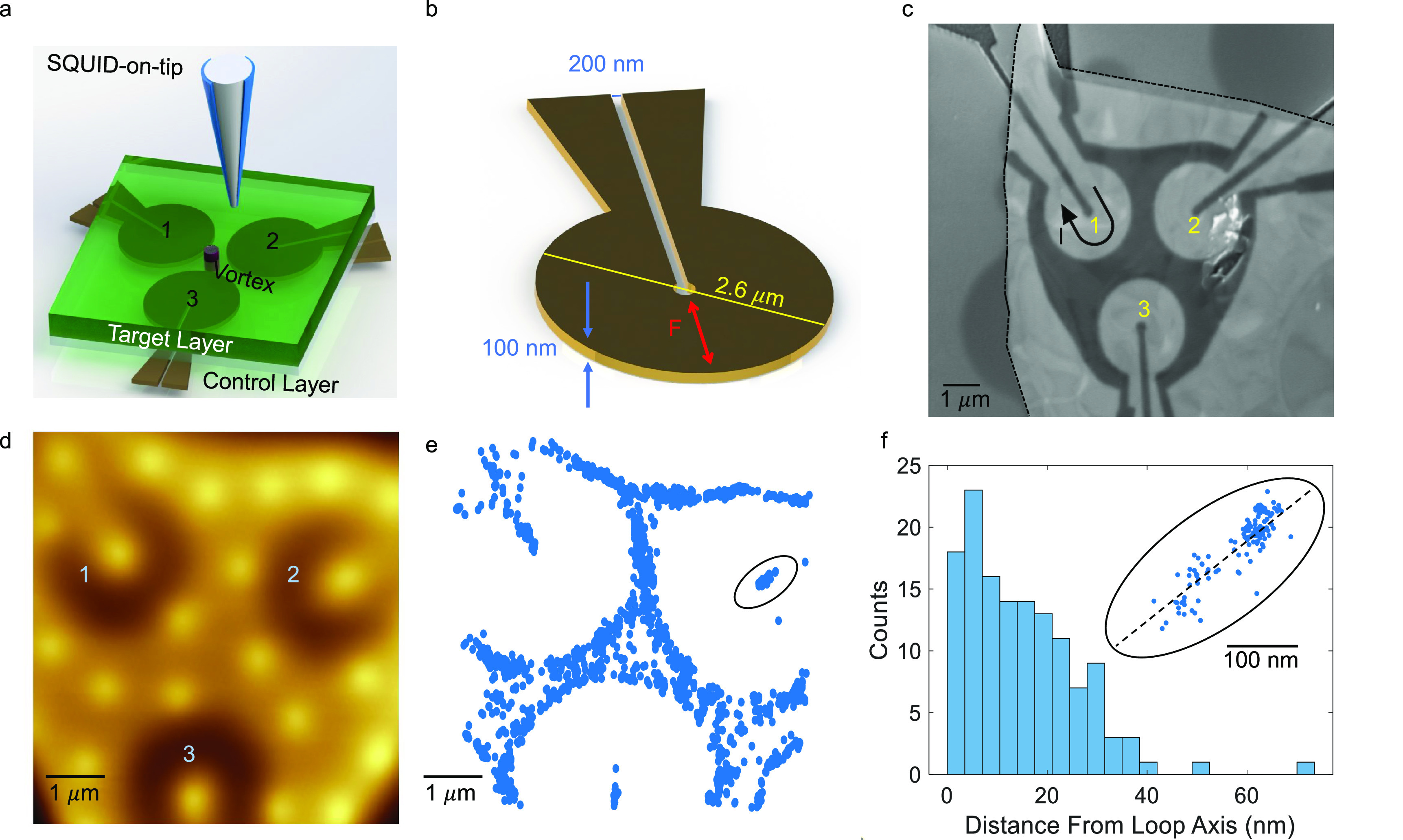
Device parameters. (a) Schematic drawing of the device. The Nb loops (gold) that are etched in the control layer are separated from a ∼50 nm thick NbSe_2_ target layer (green) by a ∼15 nm thick Al_2_O_3_ layer (transparent). A vortex is illustrated within the target layer (purple cylinder). The SQUID-on-tip (SOT) probe is represented above the device as it images the device. (b) Loop geometry. The inner diameter is 200 nm, and the thickness is 100 nm. The outer diameter is 2.6 μm (yellow line). The direction of the total force (*F*) applied by the loop on the vortex is marked by the red arrow and is parallel to the slit. (c) A top view SEM image of the device. The loops are numbered for future reference. The NbSe_2_ flake covers all three loops and is highlighted by the black dashed line. The NbSe_2_ is thin enough to be transparent to the electron beam. The curved arrow indicates the current direction running through Loop 1. (d) Field cooled state with high vortex density. All three loop centers are occupied, each by a single vortex, while no vortices reside overlapping the loop conductor. (e) Compilation of 2370 observed vortices in 500 images. The dots within the black ellipse are used to create the histogram in panel f. (f) Histogram of the points within the black ellipse in panel e. This histogram counts the distances of vortices captured within Loop 2, from the loop axis (dotted black line in the inset where a zoomed in scatter is displayed), resulting in a standard deviation of ∼25 nm.

Upon application of a current *I* through any one of the loops, a local magnetic field is generated around its center. This field interacts with the vortex via the Lorentz force **F** = *∫*(**j** × **B**) d^3^*x*. Here **B** is the magnetic field applied by the loop and **j** is the current density around the vortex core (see Note 3 in the SI). Importantly, the direction of the net force applied upon the vortex is sketched as a red arrow in Figure 1b. We orient all the loops with their force vector pointing toward the central region between them.

In Figure 1d, we present a SOT magnetic image of the device following a local field cool at 1.4 mT. To do this, the field is applied while imposing *I* > *I*_c_ in any of the loops (*I*_c_ being the critical current of the loop). This way, the dissipative current heats the target layer above its critical temperature allowing vortex entry as the loop current is lowered below *I*_c_, and the target layer cools. The image which exhibits a scatter of multiple vortices reveals two important features. First, the image shows a vortex at the center of each of the three loops. This is reproducible throughout our measurements, including images taken at higher vortex densities. The reason vortices tend to favor the loop center is related to the second observation seen in the figure—namely, the existence of an exclusion zone exactly where the NbSe_2_ target layer is placed above the Nb loop. In those regions, a weaker magnetic signal is detected, and no vortices are found. The exclusion zone is created since threading a flux line through the loops requires penetrating a second superconductor.

We emphasize these observations by compiling vortex positions extracted from 500 images (Figure 1e) where each of the 2370 detected vortices is represented by a point. This compilation includes images at high vortex density, low vortex density, and images that are taken following the application of force on the vortices using the loops. Figure 1e confirms that vortices shun the exclusion zone. The outlying cases where vortices are found within the exclusion zone represent ∼0.1% of all detected vortices.

Our design confines the vortices to a very small and elongated region aligned with the loop axis and located near the loop center. A zoomed-in scatter appears in the inset to Figure 1f, where the main panel depicts a histogram of the distances from Loop 2 axis for all the vortices detected within a 100 nm radius of its center. We find the vortices scatter within a standard deviation of ∼25 nm from the axis. Such reliable control over vortex positioning, confirmed by the uniquely high spatial resolution of the SOT, is important for future vortex-control protocols.

We now turn to the use of the Nb loops in elementary vortex manipulation. The application of current will exert an attractive or repulsive force between the loop and the vortex, depending on the current direction. We show here that we can pull a vortex into the center of a given loop, and push a vortex away from that loop. To reduce vortex–vortex interactions, we initialize the system with low vortex density. In Figure 2a we see the initial state where a vortex is centered within Loop 1. We then apply a current of *I* = 2.2 mA through Loop 1 (lower than *I*_c_ ∼ 15 mA). In Figure 2b we image the state of the system following this operation. The vortex that was previously centered within Loop 1, was pushed away from the loop, and is now localized at a pinning site in the central area between the loops. We thus coin the application of a positive current as a *push* operation. Push operations deoccupy the loops even at high densities, since loop-induced forces are far greater than vortex–vortex interactions on a length scale of 1 μm.

**Figure 2:**
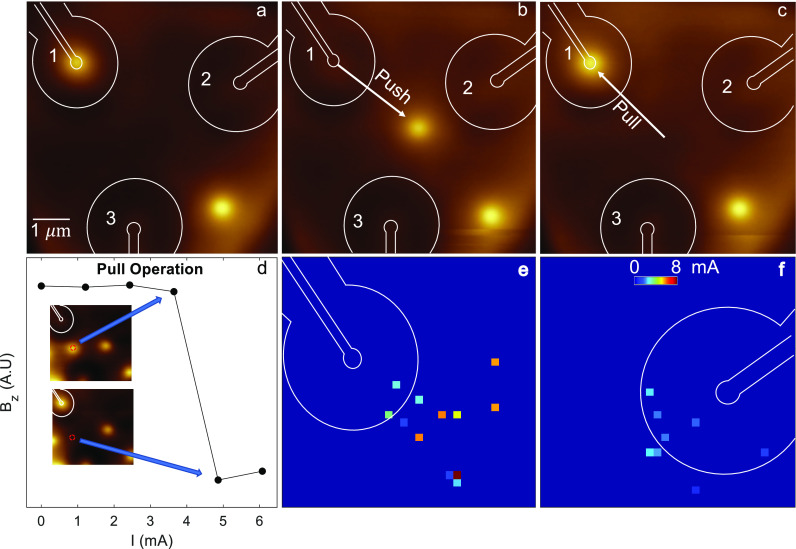
Basic vortex manipulation. (a) Initial state of the system: Loop 1 is occupied. (b) After passing 2.2 mA through Loop 1, the vortex has escaped out of the loop and is located on a pinning site. (c) After applying −14.6 mA through Loop 1, the vortex was pulled back into the loop. (d) Example of a minimal pull current measurement. The tip position is marked above a specific vortex (red circle). The current through the loop is set to each value and subsequently set to zero. The tip data (local field) is measured (black dots) until the field value drops; a scan verifies that the vortex was indeed pulled into Loop 1. (e) Current map of the minimal current needed to pull vortices from the identified pinning site into Loop 1; the minimal current value increases with distance from the loop center. (f) Same as panel d for Loop 2; the color scale is shared between panels e and f.

We demonstrate the opposite, *pull* operation, by applying a negative current on the same loop. The vortex, which was pinned in the central area between the loops, is back to the center of Loop 1 (see Figure 2c). The pull operation requires a different current, depending on the pinning strength and the distance between the vortex and the loop. We determine the pull current using the following procedure: The current within a loop is set to a specific value, then set back to zero. The local field is then measured (black dots, Figure 2d) at the vortex location (red circle). At the minimal pull current, the local field at the vortex location drops. The images in the inset, taken before and after the vortex dislodged, confirm that the vortex has been pulled into Loop 1. The location of a vortex upon pulling is reproducible with a precision better than the distribution illustrated in Figure 1f since it was acquired using a broad range of vortex densities.

While calibrating the push and pull operations, we typically acquire an image of the loop area after each current increment in the loops. For more sophisticated protocols (below) we station the SOT at the vortex location, and continuously monitor its signal until we find that the vortex has moved. We repeat this procedure to determine the minimal pulling current required to dislodge vortices residing at different locations. In Figure 2e,f, we show a map of these minimal currents (color coded) needed to pull a vortex from each pinning site to Loops 1 and 2, respectively. These statistics demonstrate that each loop has a different operational pull range, likely a consequence of variations in the loop design. Important for what follows, is that each loop can pull vortices far enough to realize inter-loop vortex shuttling operations.

We now discuss the combination of elementary push and pull operations to perform more complex protocols, including shuttle, braid and wind operations. A movie of the complete wind operation may be found in the Supporting Information. The initial state, consisting of two vortices in the field of view, created by field cooling is depicted in Figure 3a. A subsequent pull operation with Loop 3 using *I* = −2.3 mA results in the configuration appearing in Figure 3b, where “Vortex A” resides in Loop 1, and “Vortex B” resides in Loop 3. From this initial state, we apply a sequence of operations in the order listed in Figure 3r. For each operation a current pulse of a few seconds was applied. We note that, in principle, this could have been fully automated and performed much faster as we discuss below. Subsequent to every operation, the state of the system is imaged in Figure 3b–p. From now on, in order to track both vortices simultaneously we track their path using white (Vortex A) and yellow (Vortex B) dotted arrows. In Figure 3c, Vortex A is pushed from Loop 1 using 3.2 mA. In the picture, the vortex is located on a pinning site in the central area between the loops. In the table in Figure 3r, when a vortex resides in such a central pinning site it is designated as “center”. Next, we pull Vortex A into Loop 2 using *I* = −2.6 mA in Figure 3d. At this point, Vortex A has been guided from Loop 1 to Loop 2 using successive push and pull operations. We coin this a *shuttle* operation.

**Figure 3:**
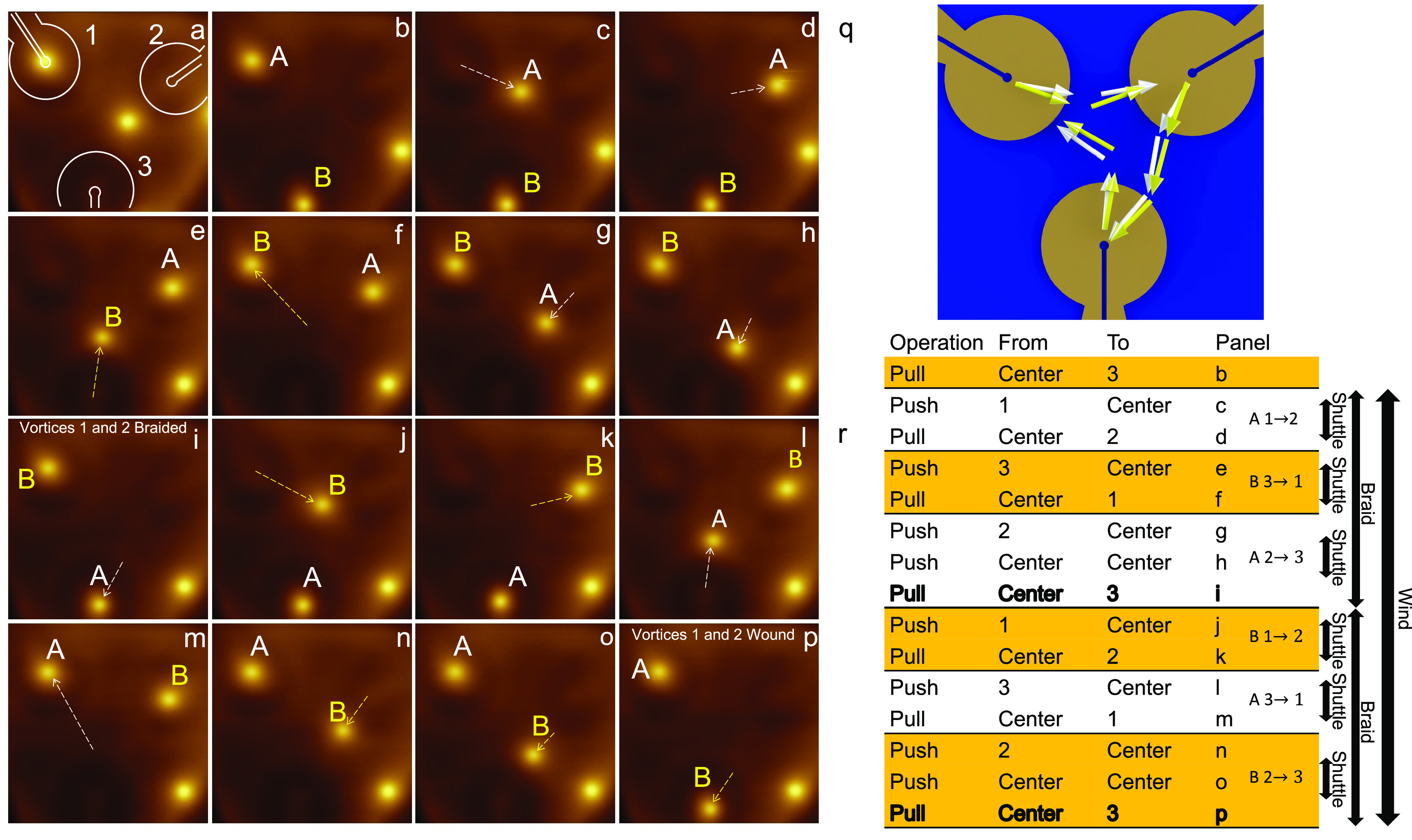
Vortex braiding. (a) Initial state of the system after a field cool down using the loop magnetic field. (b) System initialized by pulling Vortex B into Loop 3. Loops 1 and 3 are populated, prepared for the vortex winding operation. Panels b–p are used to create the movie found in the Supporting Information. (c–p) Push and pull operations, circling Vortices A and B around each other. (i) The two vortices have switched positions and have thus been braided. (p) Vortices have completed the full circle around each other, and have thus been wound. The detailed push and pull protocols are described in panel r. Each image is 6 × 6 μm^2^, contains 128 pixels, and took ∼4 min to acquire. (q) Schematic of the vortices’ full route (A, white; B, yellow). (r) Table listing the operations performed in panels a–q. Each line includes an operation (push/pull), the initial and final locations (e.g., into Loop 3, from the central area), and the panel where the outcome is imaged. Horizontal lines highlight complete shuttle operations, and bold text highlights a complete braiding operation.

In the next few steps, Vortex B is shuttled from Loop 3 to Loop 1 (Figure 3e,f), and subsequently Vortex A is shuttled from Loop 2 to Loop 3 (Figure 3g–i). The state depicted in Figure 3i, reflects a full exchange of the two vortices, i.e., a braiding operation (noted by an arrow in Figure 3r). The next sequence of operations, ending in panel p, completes a winding operation (noted by an arrow in Figure 3r). Thus, by a sequence of push–pull operations, we have demonstrated the capability of our device to carry out shuttle, braiding and winding operations—all of which are considered as fundamental in the toolbox of vortex-based quantum computation protocols.^14,29^

We now estimate the maximum operation frequency of such devices. The fundamental limiting factor is the viscous drag η_0_ and the driving force that determines the vortex speed through *F*_d_ = η_0_*v*.  kg/m s is estimated from the Bardeen–Stephen model^30^ where Φ_0_ is the flux quantum, ξ is the coherence length, and ρ_*n*_ is the normal state resistivity. This model proved to be a good approximation even at high speed.^31^ The driving force is estimated from the force curve (see Note 3 in the SI). The vortex speed *v* is calculated numerically along a straight line from the central area to the center of a loop. The average speed is 4.5 km/s. Such high velocity, comparable with the depairing velocity (∼16 km/s) is consistent with previous observations.^31^ According to our calculation, the time-of-flight between two loops is of the order of 0.3 ns, limiting the operating frequency to about 3 GHz. This frequency can be further increased if the device dimensions are shrunk.

For a fully integrated device, the vortex-control loops should operate in tandem with local vortex detection—done either using tunneling,^32^ or using RF local vortex detection. The latter could be realized using a local SQUID,^33^ fabricated above the loop center, or by using the vortex control loop as part of a pick-up loop which serves as part of a SQUID. As we show in Note 4 in the SI, the loop itself is sensitive to the presence of a vortex—as seen in the *V*(*I*) characteristics of the loop by a small variation in *I*_c_.

Our results raise the question of coherent vortex motion, and whether vortices are moved adiabatically. Taking the velocity calculated above, we propose that the condition for adiabatic motion is keeping *hv*/ξ smaller than a characteristic excitation energy scale. Which energy scale should be taken here, though, is an open question—whether it is Δ^18^ (∼1.26 meV for NbSe_2_^34^), or Δ_CdGM_—the gap between Caroli-de-Gennes Matricon (CdGM) states bound to the vortex core. The condition *hv*/ξ < Δ originates from the constraint that vortex motion does not break cooper pairs, while *hv*/ξ < Δ_CdGM_ comes from the requirement to keep the system at its ground state. Although low-lying excitations, which retain the overall parity, may not disrupt adiabatic vortex transport,^35^ it is not clear if this holds for dense CdGM spectra. For our device *hv*/ξ ≈ 3 meV at maximal speed and assuming ξ = 7 nm—at the order of magnitude of Δ, but far greater than Δ_CdGM_ in NbSe_2_. Such protocols may hence be tested on materials with a sparse CdGM spectrum, such as FeSe_0.5_Te_0.5_, where Δ_CdGM_ ≈ 100 μeV.^36^ In FeSe_0.5_Te_0.5_, we estimate η_0_ ≈ 5 × 10^–7^ kg/m s,^37^ leading to *hv*/ξ ≈ 1.5 meV. This suggests that in FeSe_0.5_Te_0.5_, vortex motion may very well be adiabatic. Moreover, the vortex speeds could be further lowered by optimizing loop geometry. Such concepts remain to be tested in future experiments.

It is also interesting to consider magnetic skyrmions, which have recently attracted attention within the spintronics community due to their potential applications.^38−40^ Our technique could be used to manipulate these magnetic textures as has been suggested in^41^ and done with MFM,^42^ due to the fact that magnetic force applied by the loops should behave the same. The exclusion zone mentioned in this paper should also be present with skyrmions and with any magnetic entity. Finally, our method should be compatible with hybrid structure that involves skyrmion-vortex pairs.^43^

## Methods

### Scanning SQUID-on-Tip Microscopy

The SOT was fabricated using self-aligned three step thermal deposition of Pb at cryogenic temperatures, as described in ref (27). The quartz tube was pulled to create a tip diameter of roughly ∼250 nm and the carefully adjusted deposition thicknesses resulted in a SQUID with a critical current of 165 μA at zero field. The relatively large diameter tip allows for high magnetic field sensitivity and a slight asymmetry in the Josephson junctions shifts the interference pattern of the SQUID, resulting in finite magnetic field sensitivity at 0 applied field.^27^ This is crucial in order to conduct the experiment at low enough field to avoid overcrowding the sample with vortices.

### Loop Fabrication

100 nm Thick Nb was sputtered on a Si/SiO_2_ chip. The loops were written using standard E-beam lithography techniques on 950-A5 PMMA. After development, the Nb was etched using reactive ion etching (RIE). After the etching process the Nb layer was coated with 15 nm Al_2_O_3_ by ALD at which point the NbSe_2_ flake was transferred on top.

## Supporting Information Available

Movie of 2d vortex winding as demonstrated in Figure 3 (MP4)
Device AFM image, design considerations, calculation of the force applied by the loops, and a demonstration of the loops acting as vortex sensors (PDF)
