# Supplementary material for: Chip-Integrated Vortex Manipulation
Source: Nano Lett. 2023 Mar 14;23(10):4669–74. doi: 10.1021/acs.nanolett.3c00324 (PMC10214491; doi:10.1021/acs.nanolett.3c00324)
Supplement: Supplementary file 2 — nl3c00324_si_002.pdf [file nl3c00324_si_002.pdf]

Supplementary Material: Chip-Integrated Vortex Manipulation

Itai Keren,[†] Alon Gutfreund,[†] Avia Noah,[†] Nofar Fridman,[†] Angelo Di Bernardo,[‡] Hadar Steinberg,[†] and Yonathan Anahory[*,†]

†*Racah Institute of Physics, The Hebrew University, Jerusalem 91904, Israel*

‡*Department of Physics, University of Konstanz, Universitätstrasse 10, 78457 Konstanz*

E-mail: yonathan.anahory@mail.huji.ac.il,hadar@phys.huji.ac.il

Supplementary Note 1. Device Details

We measure the flake thickness to be 50 nm using an AFM. The scan was taken after the measurements. The measurements ended when the tip crashed on the sample surface, its debris is visible in the image. The flake is flat in the region of interest of the experiment.

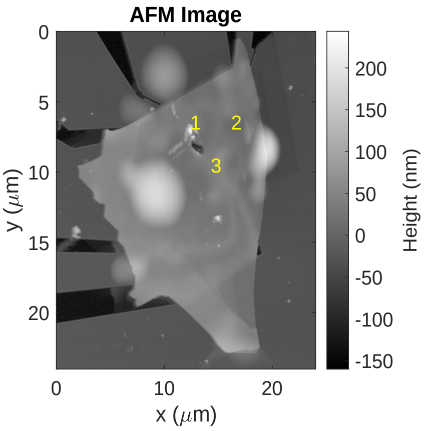

Supplementary Figure 1: AFM image of the $NbSe_2$ flake. The flake resides above the loop array. Debree from the crashed tip is visible to the left of loop 3. The flake is flat in the region of interest.

Supplementary Note 2. Design Considerations

To reach an effective localization and control of vortices, required for sophisticated shuttling and winding protocols, we had to take into account several design considerations. First, the inner diameter should be comparable to λ in order to prevent multiple vortices from entering the loop but larger than ξ to accommodate a vortex inside. The outer diameter should be large to enlarge exclusion zone but not too large on the scale of λ to avoid vortices entering the loop. We found that 200 nm for the inner diameter and 2.6 μm for the outer diameter are satisfactory parameters. The distance between different loops depends on the distance over which the force decays. This is set by the width of the loop $(r_{out} - r_{in})$. The control layer should be thicker than the target layer in order to increase the contrast in energy cost of locating a vortex in the exclusion zone. The loop thickness should not be much thicker than the target layer, to keep the target device planar. The control layer material should be a superconductor with λ as small as possible to enhance the exclusion zone effect. Smaller λ or larger superfluid density has the added benefit of enhancing the critical current of the loops which will enhance the maximal force applied on the vortices. The boundary between the two superconductors might also influence the device. We chose $NbSe_2$ as a target material because it allows us to transfer a flake on a non-planar control layer. The exfoliated relatively thick $NbSe_2$ flake remained planar which allow vortex manipulation. It might be better to revert the order when the target layer is not a van der Waals material to ensure smooth boundary between materials. The loops have to break symmetry in one axis, as shown in Fig. 1b, so the net force will point along the line of symmetry justifying the fact that all the loop leads points toward the central area in Fig. 1a and c. Finally, the target layer should be a material with relatively low pinning forces to allow the vortex manipulation.

Supplementary Note 3. Calculation of The Force Exerted By The Loop

We calculate the force applied on the vortex by the loop, by numerically solving the integral $\mathbf{F} = \int(\mathbf{j} \times \mathbf{B})d^3x$. We assume a homogeneous current density $\mathbf{j}$ within the loop while accounting for the loop shape. The magnetic field is given by the following expression $B_z = -\frac{\Phi_0}{2\pi\lambda^2}K_0(\frac{\sqrt{(x-d)^2+y^2}}{\lambda})$ - where d is the distance between the loop center and the vortex core. Here - Φ_0 is the flux quantum, λ the penetration length and K_0 is the MacDonald function. We plot the results for the largest obtained supercurrent (14 mA) in Supplementary Fig. 2a. We note that this model is only valid when the vortex is far from the loop, and the loops Meissner screening is small. The loop outer radius is marked in Supplementary Fig. 2a as a dashed line. From this force we can deduce the vortex speed, through $F_d = \eta_0 v$ (Supplementary Fig. 2b). Where $\eta_0 = \frac{\Phi_0}{2\pi\xi^2\rho_n}$ is the Bardeen-Stephen viscosity[1]. Here we used $\xi = 7$nm[2] and $\rho_n = 0.025$ mΩ·cm[3]. We calculate the position vs time curve displayed in Supplementary Fig. 2c.

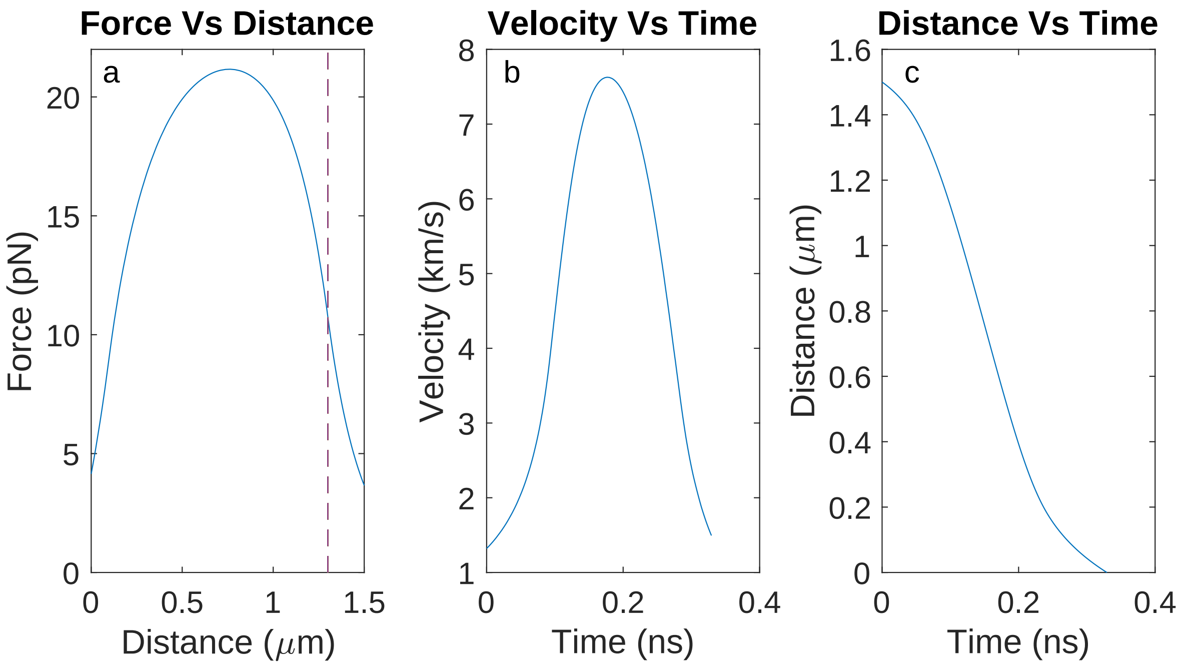

Supplementary Figure 2: Calculation of the vortex motion. **a**. Force vs Distance. This curve is simulated for a homogeneous current distribution within the loop, with the loop's symmetry broken along the axis connecting it's center to the vortex. **b**. Velocity vs time. We calculate the velocity vs time curve, by propagating the vortex through small time steps and querying the force vs distance curve for the velocity value at each position, through the Bardeen Stephen relation. **c**. same calculation as b, but the distance curve is displayed.

Supplementary Note 4. Loop as a Vortex Detector

Here, we measure the loop's I_c dependence on the presence of a vortex in the loop (occupation state). In Figure 3a we present two I-V curves (2-probe with a 100 Ohm resistor in series) of loop 1. The black curve represents the occupied state measurement while the red curve represents the unoccupied measurement, both measurements are performed with the same polarity - with voltage ramping up from zero. One can see a difference in critical current values (I_c) at which resistance of the loop becomes finite. We repeat this measurement many times and draw three histograms In Figures3b-d. Surprisingly, we note that I_c increases when the loop is occupied. We attribute this effect to the magnetic flux being focused at the center of the ring, reducing the effective field on the loop and

therefore increasing I_c. It should be noted that this method of sensing is invasive since the large current also generates a force on the surrounding vortices. Also, this measurement requires crossing the critical current, introducing heat to the system. Nevertheless, if one applies a pulling force and find the loop to be unoccupied, one can conclude that the loop was not occupied even prior the the readout.

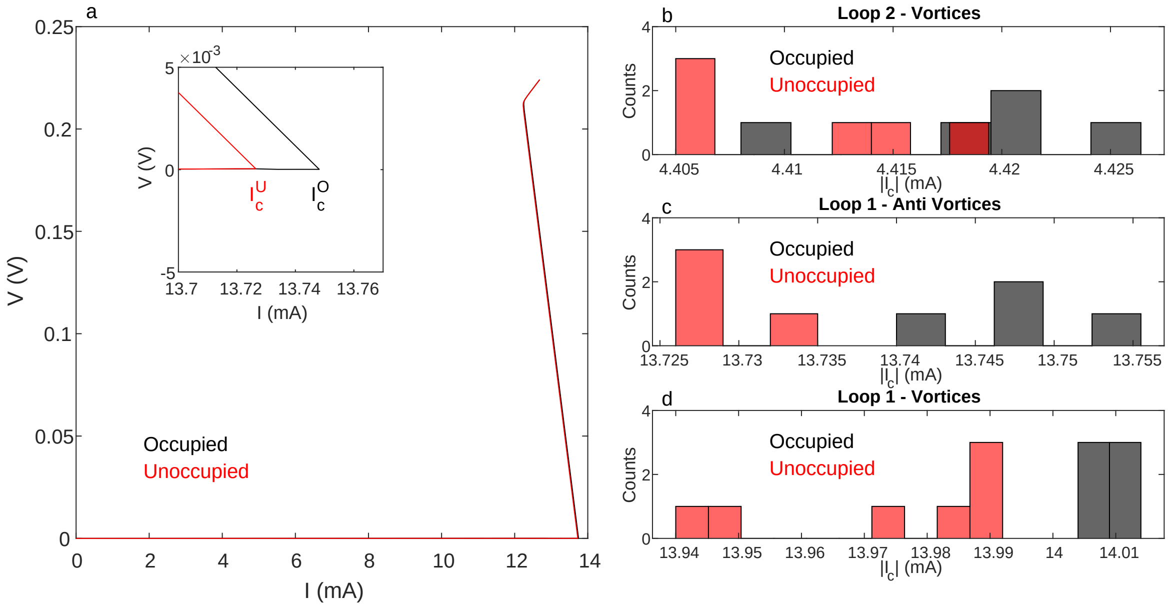

Supplementary Figure 3: **Critical current of the loop as a function of its occupation state a**. Typical I_c measurement for an occupied (black curve) and unoccupied (red curve) loop 1. **b-d**. A histograms of measurements on loops 1 and 2, including vortices **b-c** and anti-vortices **d** representing two orientations of the the external field. Notably, the critical current varies by 20 μA depending on the occupation state of the loop.

References

(1) Bardeen, J.; Stephen, M. J. Theory of the motion of vortices in superconductors. *Physical Review* **1965**, *140*, A1197.

(2) Nader, A.; Monceau, P. Critical Field of 2H-NbSe$_2$ Down to 50mK. *SpringerPlus* **2014**, *3*.

(3) Bawden, L. et al. Spin–valley locking in the normal state of a transition-metal dichalcogenide superconductor. *Nature Communications* **2016**, *7*.
